# Fatigue behavior of sintered, glazed and glass-infiltrated surfaces of 5Y-PSZ bonded plates

**DOI:** 10.1590/1807-3107bor-2024.vol38.0027

**Published:** 2024-12-09

**Authors:** Ana Carolina da Silva, Laura Patrícia Nadal Ortiz, Larissa Márcia Martins Alves, Kiara Serafini Dapieve, Tiago Moreira Bastos Campos, Marco Antonio Bottino, Gilmar Patrocínio Thim, Luiz Felipe Valandro, Renata Marques de Melo Marinho

**Affiliations:** a Universidade Estadual Paulista – Unesp, Institute of Science and Technology, Department of Dental Materials and Prosthodontics, São José dos Campos, SP, Brazil.; b Universidade de São Paulo – USP, Bauru School of Dentistry, Department of Prosthodontics and Periodontology, Bauru, SP, Brazil.; c Universidade Federal de Santa Maria – UFSM, School of Dentistry, Program in Oral Science, Santa Maria, RS, Brazil.; d Instituto Tecnológico de Aeronáutica – ITA, Physics Department, São José dos Campos, SP, Brazil.

**Keywords:** Dental Restoration Failure, Zirconium, Ceramics, Mechanical Tests

## Abstract

This study evaluated the effect of different occlusal surface finishes (glaze and silica glass infiltration) on surface characteristics and fatigue behavior of partially stabilized zirconia (PSZ) plates adhesively bonded onto epoxy resin discs. PSZ disc specimens (n = 15; Katana blocks STML, Kuraray Noritake Dental) were produced (Ø = 10 mm; thickness = 1.2 mm) and allocated into 3 groups: As sintered (S), silica glass infiltration (SGI), and glaze application (G). The PSZ intaglio surface was air-abraded with 50-µm alumina powder followed by bonding agent application. All produced PSZ were adhesively cemented onto dentin analogue discs made of epoxy resin material (Ø = 10 mm; thickness = 2 mm). Step stress fatigue test was performed (load ranging from 200 to 1800 N; step size 100 N and 10,000 cycles; 20 Hz). The topographic, microstructural, and fractographic analyses were performed by scanning electron microscopy. Results: No statistically significant difference in fatigue behavior was detected among the groups. All failures started at the bonding surface. Silica glass-infiltration and glaze layer application provided a smoothing effect, while the sintered group had a surface with grooves. The occlusal surface finishing method (silica glass infiltration or glazing) had no deleterious effect on fatigue behavior of adhesively bonded PSZ plates.

## Introductions

The continuous search for improvement of the optical properties of 3Y-TZP zirconia has led to the development of structural and compositional changes of this ceramic by the dental market, aiming to increase translucency by decreasing the alumina amount and eliminating porosity by increasing sintering temperatures. This resulted in a modest improvement in translucency, allowing its use as a monolithic restoration, but still not enough to meet the esthetic requirements for anterior rehabilitations.^1^

Advances in research enabled the development of a new, less opaque zirconia generation.^2^ This improvement was achieved by increasing the yttria amount to 4 mol% (4Y-PSZ) or 5 mol% (5Y-PSZ), resulting in partially stabilized zirconia (PSZ).^3^ The higher yttria content increased the amount of non-birefringent cubic phase. Therefore, light transmition was improved as isotropic cubic grains decrease light scattering from grain boundaries and other microstructural defects. However, the mechanical properties were affected since the cubic phase does not undergo transformation toughening.^1,4^ As part of this new generation, multi-layer materials were introduced to the dental market to better mimic enamel and dentin shades. Multi-layered zirconias then consist of several layers with color gradations (35% in the enamel, 15% in the first gradation layer, 15% in the second gradation layer and 35% in the dentin).^5^

In order to improve the mechanical performance of partially stabilized zirconia, silica glass infiltration was proposed. This method resulted in a graded silica glass/zirconia layer with a relatively lower elastic modulus than pure zirconia. This graded structure improved flexural strength by ∼ 70% compared to non-infiltrated zirconia, providing a durable resin cement bond without compromising the translucency of 5Y-PSZ.^6^ The graded layer between glass and zirconia decreases the surface elastic modulus and transfers the stresses to the subsurface.^6-9^ In addition, studies developed by Campos et al.^10^ and Silva et al.,^11^ showed that the coefficient of thermal expansion of the infiltration glass is similar to zirconia. This compatibility favors the compressive residual stresses acting against crack propagation, which increases the mechanical behavior of the set.^11^

A previous study showed that although glaze application and/or polishing provide different finishes, neither one affects the fatigue strength of a zirconia occlusal surface tested under compression.^12^ However, there are no reports in the literature on how a 5Y-PSZ zirconia graded with silica glass behaves under compressive forces. Therefore, it is important to know the behavior of such alternative finish, since it not only provides a glass surface, but also generates an elastic modulus gradient on the material,^13,14^ which increases the damage resistance of zirconia.^15^

Thus, the effect of different finishes on the surface of zirconia polycrystals with high cubic phase subjected to intermittent fatigue cyclical loading should be investigated. This study aimed to evaluate the effects of different occlusal surface finishes (silica glass infiltration and glazing) on fatigue and surface characteristics of partially stabilized zirconia (PSZ) plates adhesively bonded to dentin analogs. The anticipated null hypothesis was that the distinct occlusal surface finishes do not affect the fatigue strength of PSZ.

## Methodology

The materials used in this study with their respective composition, manufacturers, and batch numbers are described in Table 1.

**Table 1:** Description, firing cycles, coefficient of thermal expansion (CTE), and elastic modulus (E) of materials used in the study.

Materials	Composition	Manufactures	Batch number	Firing cycle	CTE (K^-1^)	E (Gpa)
Zirconia PSZ	ZrO_2_ + HfO_2_ – 88- 93%, Yttrium oxide (Y_2_O_3_) – 7-10%, other oxides – 0-2%.	Katana Super Translucent Multi Layered, STML, Kuraray Noritake Dental, Miyoshi, Japan	DTGUV	initial temperature, 25°C; heating time, 17 min; temperature elevation rate, 80 °C/min; final temperature, 1550 °C; and dwell time at the final temperature, 120 min.	10,17.10^-6 16^	274.90^16^
Infiltration glass	SiO_2_ −68%, Al_2_O_3_ −11.7%, CaO 3.0%, Na_2_O −7.3% and K_2_O −10.0%	Developed by Moreira Bastos Campos et al., 2021	–	*	10,0.10^-6 11^	77.22 ^10^
Propylene glycol solution	C_3_H_8_O_2_	Labsynth, Diadema, São Paulo, Brazil	178730	–	–	–
Glassy-based material applied by spray	Body stains - special low fusing glaze material to create a silky matte and sealed surface	Vita Akzent, VITA Zahnfabrik, Baden-Württemberg, Germany	A0764	initial temperature, 500°C; heating time, 9 min; temperature elevation rate, 80 °C/min; final temperature, 900 °C; and dwell time at the final temperature, 1 min.	11,3.10^-6 17^	82.5 ^10^
10% hydrofluoric acid	< 10% hydrofluoric acid	FGM Dentscare Ltda, Joinville, Brazil	110718	–	–	–
Dual cement	Dimethacrylate, HEMA, iterbium trifluoride and spheroidal mixed oxides	Multilink N, Ivoclar Vivadent, Schaan, Liechtenstein	Y06983			4.9^18^
Primer	HEMA phosphonic acid methacrylate monomers	Multilink A and B, Ivoclar Vivadent	Multilink A: Y25800 Multilink B: Y31808	–	–	–
Silane	Alcohol solution of silane methacrylate, phosphoric acid methacrylate and sulphide methacrylate	Monobond N, Ivoclar Vivadent	Y33681	–	–	–
Epoxy resin	Continuous filament woven fiberglass bonded with epoxy resin.	Carbotec GmbH & Co. KG, Konigs, Wusterhausen, Germany	–	–	8,0.10^-5 19^	14.9^20^

* A single firing cycle for zirconia sintering and glass infiltration was performed.

### Specimen preparation

Katana STML blocks (Super Translucent Multi Layered, Kuraray Noritake Dental, Miyoshi, Japan) were rounded in a polishing machine (EcoMet/AutoMet 250, Buhler, USA) in order to obtain a cylindrical shape (Ø = 10 mm). Next, 1.6-mm-thick discs were cut on a precision cutting machine (IsoMet 1000 Precision Saw, Buehler, USA) and subsequently finished in a polishing machine (EcoMet/AutoMet 250) with a sequence of #600-#1200 abrasive wet paper to remove the irregularities of the cut. Then, the specimens were randomly allocated into 3 groups (n = 15) according to the surface finish: (S) Sintered – control group, (SGI) Silica Glass-Infiltrated, and (G) Glazed.

### Glass preparation

Glass powder was made by the sol-gel method. The silica source was silicic acid obtained through the passage of a 10% w/w aqueous sodium silicate solution (Labsynth, São Paulo, Brazil) by an ion exchange resin (IR120, Dow Corning, Midland, USA). After synthesizing, the silicic acid (0.5 mol/L) was mixed with aluminium nitrate (Labsynth), calcium nitrate (Labsynth), sodium nitrate (Labsynth), and potassium nitrate (Labsynth) to obtain the following composition of mass oxides: 68% SiO_2_, 11.7% Al_2_O_3_, 3.0% CaO, 7.3% Na_2_O, and 10.0% K_2_O. This mixture was placed in an oven at 100°C for 24 h, and then the material was calcinated in an oven for 5 h at 650°C. After calcination, the resulting material was ground and sieved (200 mesh).^10,21^

### Occlusal surface finishes

#### As sintered (S)

The sintered group was composed by previously prepared samples as explained in section Specimen preparation, without any treatment on the occlusal surface of the specimens.

#### Silica glass-infiltrated (SGI)

First, 1 g of silica glass powder was mixed with 0.23 g of Propylene Glycol P.A.- A.C.S. until a homogeneous slurry was obtained. Then, the mixture was applied on the occlusal surface to the pre-sintered specimen with a fine-tip brush. Secondly, the specimens of all groups were sintered in a specific furnace (Infire HTC Speed, Dentsply Sirona, Bensheim, Germany) at 1,550°C for 2 h, as recommended by the manufacturer. Thus, the final dimensions of the glass infiltrated discs were thickness of 1.28 mm (± 0.05) and Ø of 10 mm, due to 20% shrinking after total densification of the material.

#### Glaze (G)

Low-fusing glaze spray (VITA Akzent Plus, VITA Zahnfabrik, Bad-Sackingen, Germany) was applied twice for 5 s each time at a distance of 10 cm and fired in a vacuum furnace (Vita Vacumat 6000, Vita Zahnfabrik) following the protocol recommended by the manufacturer.

#### Roughness analysis

The specimens from each group (n = 10) were analyzed in a contact profilometer (SJ 400, Mitutoyo, Tokyo, Japan). Three equidistant parallel measurements were performed on each specimen at a speed of 0.2 mm/s, and three other parallel measurements were performed after the same specimen was rotated 90 degrees. The analysis was performed following ISO 4287-1997, with a Gaussian filter and a cut-off wavelength value of 0.8 mm. Average values were calculated for each sample, and the mean Ra (average roughness) and Rz (ten-point-mean roughness) values were submitted to statistical analysis.

#### Final sample set

Chen et al.^22^ developed a three-layer configuration to emulate an occlusal restoration for a posterior tooth. Epoxy resin discs (Carbotec GmbH & Co. KG, Konigs, Wusterhausen, Germany) were used as a substrate in a plate format. An epoxy resin cylinder (70 mm high) was sectioned using a precision cutting machine (IsoMet 1000 Precision Saw) to obtain discs with a final thickness of 2 mm in and a diameter of 10 mm.

#### Conditioning and cementation procedures

The surfaces opposite of the glaze or silica glass-infiltration finishes were sandblasted (Sandblaster III Trijato Goldline, Sao Paulo, Brazil) with 50-µm aluminum oxide particles (Bioart Ltda., São Paulo, Brazil) for 30 s at 0.2 MPa. The epoxy resin surfaces were subsequently etched with 10% hydrofluoric acid (FGM, Joinville, Brazil) for 30 s. Next, both the zirconia and epoxy resin discs were washed with water for 30 s, cleaned with distilled water in ultrasonic bath for 2 min to remove any residual acid, and gently air dried.

After drying, Multilink Primers A and B (Ivoclar Vivadent, Schaan, Liechtenstein) were mixed in a 1:1 ratio, applied on the epoxy resin surfaces with a microbrush (30 s), and gently air dried. A silane agent (Monobond N, Ivoclar Vivadent) was actively applied to the zirconia intaglio surface for 15 s, which was left undisturbed for 45 s and gently air dried.

Then, zirconia and resin epoxy discs were adhesively cemented with a resin cement (Multilink N, Ivoclar Vivadent) according to the manufacturer's instructions. The assembly was kept under a constant load of 750 g applied to the ceramic surface, promoting uniform cement spreading. The excesses of resin cement were removed with a microbrush and light-activation was performed (high intensity of 1200 mW/cm²; wavelength of 385 to 515 nm; Bluephase, Ivoclar Vivadent) for 40 s on the occlusal surface, followed by 10 s on each side of the bonded interface (0°, 90°, 180°, 270°). The specimens were stored in distilled water at 37°C for two days until fatigue testing.

#### Step-stress fatigue test

First, an adhesive tape (110 μm) was fixed to the occlusal surface of each specimen to improve contact with the piston, promoting better stress distribution and preventing damage to the contact surface, which could result in propagation of a cone crack. An additional thin film of a non-rigid material (cellophane, 2.50 μm) was placed between the piston and the sample to imporve stress distribution.^12^ Next, the specimens (n= 15) were tested by the step-stress method^23, 24^ using an electric machine (Instron Electro Puls E3000, Instron, USA). The load was applied by a 40-mm diameter stainless-steel hemispheric piston in the center of the specimens ^25^ in distilled water. An initial load of 200 N for 5000 cycles was performed to accommodate the piston/specimen set. Then, incremental loads of 100 N for 10,000 cycles starting from 400 N were applied with a frequency of 20 Hz until failure (radial cracks) of the sample. The specimens were checked for cracks at the end of each step by light oblique transillumination.^26^ The evaluated outcome was radial crack or fracture. If these failures were found the sample was considered as ‘failed’ and the collected data (load and number of cycles to failure) were recorded for statistical analysis. If the specimen survived, the load increment was increased, and the test proceeded until failure.

#### Statistical analysis

The data were subjected to survival analysis by Kaplan Meier and Mantel-Cox Log Rank test using IBM SPSS software (IBM, Armonk, USA). The survival rates relative to each testing step were also tabulated. Additionally, the Weibull moduli (shape parameter) were obtained using the Minitab 16 software program (Minitab Inc., State College, USA), under the maximum likelihood to describe structural reliability of each tested condition.

#### Topographic, microstructural and fractographic analysis

High-resolution Field Emission Gun Scanning Electron Microscopy (FEG-SEM) analysis (Magellan 400L, FEI Company, Moravia, Czech Republic) was performed (n = 1) using a secondary electron (SE) detector to determine grain size to characterize the specimens’ topography and microstructure.

One representative fractured sample per group was subsequently analyzed by scanning electron microscopy (SEM, Tescan Vega 3 model, Tescan, Czech Republic) to determine the failure origin and to characterize the fractures.

### Results

**Figure 1:**
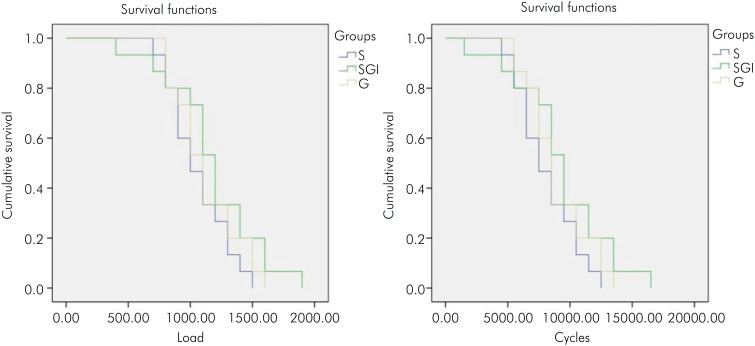
Cumulative survival plots based on load (left) and number of cycles for failure (right) obtained by Kaplan-Meier test.

**Figure 2:**
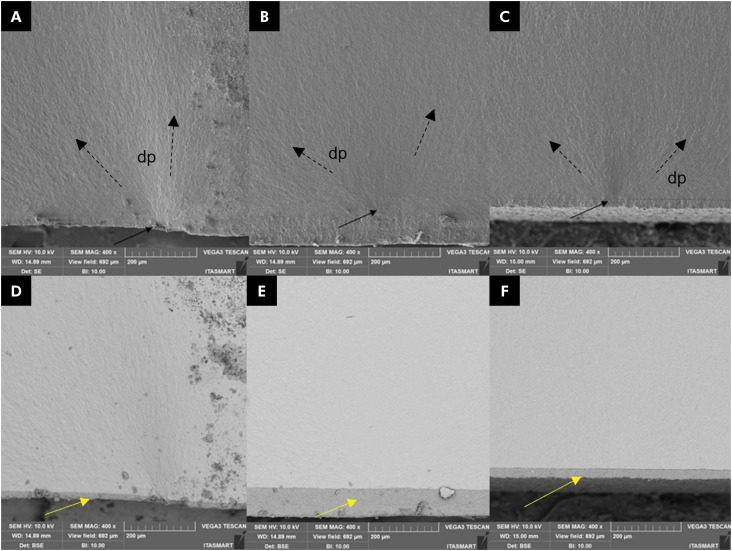
SEM images of the fracture region using secondary and backscattered electrodes. Sintered (A and B), silica glass-infiltrated (C and D), and glaze (E and F) samples. Cement layer is indicated by yellow arrows while the fracture origin is demarcated by solid black arrows. Origins were mostly on the tensile side of the specimens. The direction of crack propagation (dp) is shown by black dashed arrows.

All the specimens failed the fatigue test. According to statistical tests (Kaplan Meier with Mantel-Cox log-rank post-hoc tests; α = 0.05), none of the surface finishes affected the fatigue failure load or the number of cycles until failure (Figure 1 and Table 2). No statistical difference for Weibull moduli and characteristic strengths could be detected (Table 2). The images obtained with secondary electrons showed that all failures started at the interface between resin cement and zirconia (black arrows), whereas the images of backscattered electrodes showed the presence of cement layer (yellow arrows) (Figure 2).

**Table 2:** Mean ± standard deviation (SD) of roughness values for the Ra and Rz parameters, means and respective 95% confidence intervals (IC) of survival (Kaplan-Meier Mantel-Cox tests) and Weibull analysis.

Groups	Roughness analysis				Survival analysis		Weibull analysis	
	Ra ± SD	µm	Rz ± SD	µm	Fatigue failure load (N)	Cycles until failure	Weibull modulus-m	Characteristic strength (Mean-95% CI)
S	0.66^A^	± 0.31	4.48^A^	± 1.95	1060.00^A^ (939.33–1180.67)	81000.00^A^ (68932.90–93067.10)	5^A^ (3.39–7.37)	1154^A^ (1036 –1284)
SGI	0.23^B^	± 0.03	0.88^B^	± 0.57	1180.00^A^ (988.53–1371.47)	93000.00^A^ (73853.24–112146.76)	3.61^A^ (2.43–5.38)	1308^A^ (1129–1515)
G	0.80^A^	± 0.17	4.43^A^	± 1.20	1120.00^A^ (985.83–1254.17)	89000.00^A^ (76341.03–101658.97)	4.69^A^ (3.19–6.89)	1223^A^ (1091–1373)

Different letters on each column indicate statistical differences for each outcome (Tukey's test, p < 0.05).

Roughness results (Ra and Rz) are shown in Table 2. Silica glass infiltration had lower surface roughness, regardless of the parameter analyzed (Ra or Rz), compared to glazed and as-sintered zirconia (p < 0.001).

**Figure 3:**
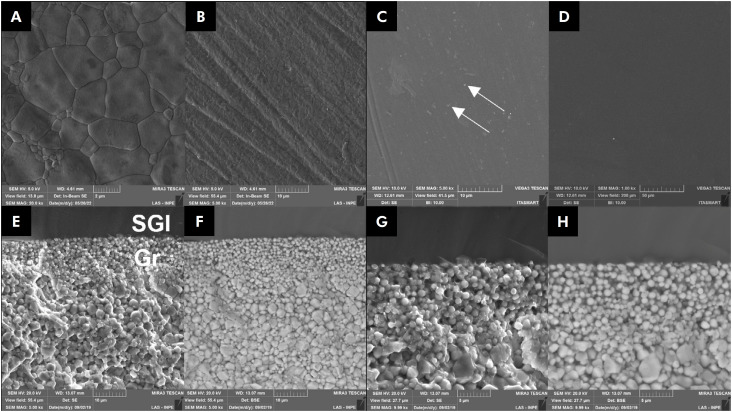
Microstructures of zirconia surfaces according to surface finish. Sintered (A), polishing (B), glaze (C), and silica glass infiltration (D). Cross-section of a silica glass-infiltrated sample (E, F, G, and H). SGI: silica glass-infiltration, Gr: graded region. Irregularities on the glaze group are demarcated by solid white arrows.

**Figure 4:**
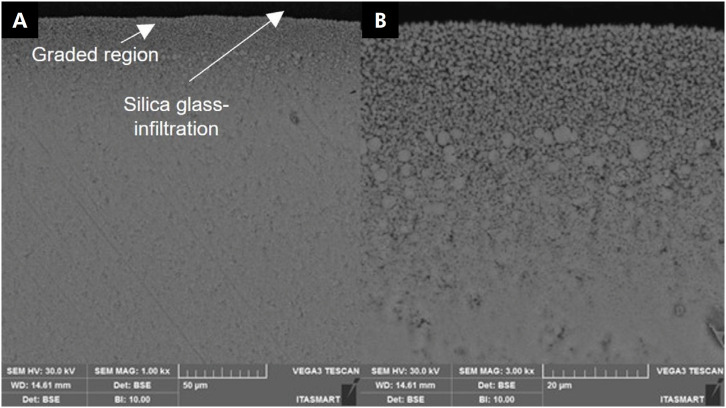
Polished cross-section of a silica glass-infiltrated sample using a backscattered electron beam. (A) - 1000x and (B) - 3000x. The graded region and silica glass-infiltration are indicated by white arrows.

The SEM analysis (Figure 3) showed different topographies among the surface conditions. Figure 3A presents as-sintered zirconia, in which two zirconia grains of different sizes can be observed (∼ 4 and 1 μm diameters), with the surface containing mostly large grains. Figure 3B) shows a surface with grooves and scratches, where the grain contours are not clearly visible. The G (Figure 3C) and SGI groups (Figure 3D) had a smoother surface in SEM analysis, with less irregularities and surface flaws. Figure 4 also presents cross-sectional micrographs of a silica glass-infiltrated zirconia performed by two detectors. Figure 3E and G show images from secondary electrodes that contain detached grains at the graded region, while backscattered electrode images (Figure 3F and D) show round and smaller zirconia grains on the surface, and larger and faceted grains on the subsurface. Figure 4 shows an image obtained with backscattering detector. Thus, pure glass (dark area) and the contour of zirconia grains (light color) can be observed. In Figure 4A (1.00kx), a graded region (∼50 µm) is shown, and in Figure 4B at a higher magnification (3000x) a grain size reduction on the surface and larger grains on the subsurface can be seen.

### Discussion

This study evaluated the fatigue behavior of different occlusal surface finishes on partially stabilized zirconia adhesively bonded plates. The sintered, glazed, or with silica glass infiltration occlusal surfaces of the bonded plates had no deleterious effect on fatigue and behaved similarly in all groups. Therefore, the null hypothesis was accepted.

The use of epoxy resin, and the occlusal surface application of glaze and infiltration glass were chosen to simulate a dental restoration. Some studies have already shown that epoxy resin is widely used as a dentine analogue to support ceramic samples due to its elastic and mechanical behavior similar to dentin (elastic modulus −14.9 GPa and Poisson's ratio- 0.31).^20,27,28^

The silica infiltration extends deep inside the subsurface, promoting an elastic moduli gradient,^6-^, with a low elastic modulus in the silica glass and a high elastic modulus in zirconia (∼210 GPa).^16,20,29^ On the other hand, surface finishes such as glaze and polishing only modify the material surface.^13^

The higher the elastic modulus of a material, the harder it is. Epoxy resin is a material that is more resistant to elastic deformation, since its modulus is lower than that of zirconia. Damage from occlusal contact can be limited to the external glass layer, and cracks are unlikely to propagate from an area with lower elastic modulus and fracture toughness to an area with higher elastic modulus and fracture toughness.^15,30^ Biomechanically, the gradual increase in elastic modulus enables a better stress distribution, since there is less stress concentration on the surface and greater stress dissipation towards the interior of the material, which increased flexural and fatigue strength.^6,14,15,31,32^ Otherwise, infiltration of silica glass in tetragonal zirconia promoted a thicker layer of around 120 μm,^15^ but only 50 µm of this layer was within cubic zirconia,^6^ which is confirmed by our findings (Figure 4). Therefore, this may explain the similar fatigue behavior of silica glass-infiltrated zirconia and both non-infiltrated conditions (Figure 1, Table 2). The thin gradation layer may not have been as effective as tetragonal zirconia gradation layers regarding stress distribution, which is consistent with a previous study.^33^

The most common failure type observed herein (as well as clinically) was radial cracking, which occurred at the cementation surface, since it is the region under tensile stress.^25,34^ Therefore, the finishes did not influence fatigue behavior, and as a result, the same failure mode was found for all groups (Figure 2). This was a consequence of the equal conditions at the intaglio surface, added to the previously mentioned fact that the silica glass layer may not improve the mechanical behavior of the infiltrated zirconia. Therefore, since the failure mode was the same for all the finishes and the intaglio surface dominated the mechanical behavior, the Weibull analysis did not detect statistical differences in Weibull moduli and characteristic strengths (Table 2).

A surface with grooves and scratches was observed on as-sintered PSZ. Although glass-infiltrated zirconia presented the lowest surface roughness, a similar surface microstructure, homogenous and smooth, was observed for glazed zirconia. The glaze and glass layers fill flaws and pores (Figure 3), decreasing irregularities.^11,35^ Moreover, the defects on the occlusal surface were mostly under compressive stresses and therefore did not induce failure/fracture of the material.^36^

The silica glass infiltration not only filled and decreased zirconia surface defects, but also changed the zirconia grain shape from faceted to round (Figure 4), as shown in a previous study.^37^ This change might reduce stress concentration, an thus improve zirconia's mechanical performance.^38^ Thus, the silica glass infiltration performed at the cementation surface would provide more effective stress distribution and improve the zirconia fatigue behavior, but this needs further investigation.

Although the groups presented similar fatigue behavior, the surface with silica glass infiltration was the smoothest (Figure 3D) (Table 2). It is well known that a rough surface increases the wear of the antagonist tooth and promotes the accumulation of microorganisms.^29,39^ This smoothing effect is important to consider because it significantly decreases microorganisms adhesion. According to Silva et al.^11^ and Ribeiro et al.,^40^ the same composition of infiltration glass showed a similar surface effect, with a low quantity of microorganisms on the glass-infiltrated surface.^11,41^

The silica glass infiltration used in this study is independent of a specific stoichiometry, which enables adding different ion concentrations for therapeutic purposes.^41^ In this way, some compositions of glass for infiltration can be formulated to meet different clinical needs, such as preventing infections (*i.e.* secondary caries). Ribeiro et al.^40^ showed antimicrobial effect against *Candida albicans, Streptococcus mutans*, and *Streptococcus sanguinis* when glass infiltration is associated with silver ions.

Limitations include the use of a simplified plate model to mimic posterior restorations (disck-disck set-up), the lack of temperature and pHs variations, and the lack of off-center movements during cyclic, and further investigation is recommended.

### Conclusion

The surface finishes (glaze and silica glass infiltration) behaved similarly in terms of fatigue effects on the bonded partially-stabilized zirconia (PSZ) plates.

Considering the test geometry (bonded discs), the fatigue behavior of the sintered, glazed, and silica glass-infiltrated samples was determined by conditions (defects, fails/voids at the interfaces or cement layer) of the bonding zone. The silica glass infiltration groups presented the smoothest surface. The silica glass infiltration group showed a graded transition between PSZ and glass infiltration.
